# Phenotypic and Genotypic Diversity of *Ascochyta fabae* Populations in Southern Australia

**DOI:** 10.3389/fpls.2022.918211

**Published:** 2022-08-02

**Authors:** Sara N. Blake, Robert C. Lee, Michelle H. Russ, Elizabeth A. Farquharson, Jade A. Rose, Herdina, Shashi N. Goonetilleke, Lina M. Farfan-Caceres, Johannes W. Debler, Robert A. Syme, Jennifer A. Davidson

**Affiliations:** ^1^Pulse and Oilseed Pathology, Plant Health and Biosecurity, Crop Sciences, South Australian Research and Development Institute, Adelaide, SA, Australia; ^2^Centre for Crop and Disease Management, School of Molecular and Life Sciences, Curtin University, Bentley, WA, Australia; ^3^Crop Improvement, Plant Health and Biosecurity, South Australian Research and Development Institute, Adelaide, SA, Australia

**Keywords:** *Didymella fabae*, ascochyta blight, *Vicia faba*, pathogenicity, pathogenicity group, mating type, DArTseq

## Abstract

*Ascochyta fabae* Speg. is a serious foliar fungal disease of faba bean and a constraint to production worldwide. This study investigated the phenotypic and genotypic diversity of the *A. fabae* pathogen population in southern Australia and the pathogenic variability of the population was examined on a differential set of faba bean cultivars. The host set was inoculated with 154 *A. fabae* isolates collected from 2015 to 2018 and a range of disease reactions from high to low aggressiveness was observed. Eighty percent of isolates collected from 2015 to 2018 were categorized as pathogenicity group (PG) PG-2 (pathogenic on Farah) and were detected in every region in each year of collection. Four percent of isolates were non-pathogenic on Farah and designated as PG-1. A small group of isolates (16%) were pathogenic on the most resistant differential cultivars, PBA Samira or Nura, and these isolates were designated PG-3. Mating types of 311 isolates collected between 1991 and 2018 were determined and showed an equal ratio of MAT1–1 and MAT1–2 in the southern Australian population. The genetic diversity and population structure of 305 isolates were examined using DArTseq genotyping, and results suggest no association of genotype with any of the population descriptors *viz*.: collection year, region, host cultivar, mating type, or PG. A Genome-Wide Association Study (GWAS) was performed to assess genetic association with pathogenicity traits and a significant trait-associated genomic locus for disease in Farah AR and PBA Zahra, and PG was revealed. The high frequency of mating of *A. fabae* indicated by the wide distribution of the two mating types means changes to virulence genes would be quickly distributed to other genotypes. Continued monitoring of the *A. fabae* pathogen population through pathogenicity testing will be important to identify any increases in aggressiveness or emergence of novel PGs. GWAS and future genetic studies using biparental mating populations could be useful for identifying virulence genes responsible for the observed changes in pathogenicity.

## Introduction

Faba bean (*Vicia faba*) is a high-protein and cool-season grain legume crop that has been cultivated throughout the world for human and animal consumption. Fresh, dry, and canned faba bean seed is a dietary staple in many parts of the world, including the Middle East and Africa. Worldwide, over 4.3 million tonnes of dry beans were produced in 2020 (FAOSTAT, 2021). Faba bean has been cultivated in Australia since 1980 and is grown predominantly in the temperate southern regions of South Australia (SA) and Victoria (VIC) where annual rainfall is 350–600 mm. Faba bean comprises approximately 10–15% of the annual Australian pulse crop and, in 2019, the area that was sown to faba bean was 196,000 hectares with 327,000 tonnes produced that year (ABARES, 2020). The major growing regions include the Yorke Peninsula, Eyre Peninsula, Mid North, and Southeast in SA, and the Wimmera and Mallee regions of VIC. There are smaller areas of production in the sub-tropical environment of northern New South Wales (NSW). Australia is the fourth largest producer and is the world’s number one exporter of faba bean. Production is valued at up to AU$230 M per year and over 90% of the Australian crop is exported, primarily to the Egyptian market for human consumption (Kaur et al., 2014; FAOSTAT, 2021).

Ascochyta blight (AB) is a polycyclic foliar disease caused by the fungal pathogen *Ascochyta fabae* Speg. (Teleomorph: *Didymella fabae*). AB is a serious constraint to production in all the major faba bean growing regions of the world including the Middle East, Europe, Canada, and New Zealand (Kaur et al., 2014). Yield loss due to AB can be as high as 90% depending on inoculum load and conditions being conducive for the disease but typically range from 35 to 40% (Hampton, 1980; Jellis et al., 1984; Paull et al., 2006; Diaz-Ruiz et al., 2009). *A. fabae* is heterothallic and compatible mating types MAT1–1 and MAT1–2, which are both present in Australia, are required for the development of fertile pseudothecia and viable ascospores (Yoder et al., 1986; Kaiser, 1997). Pseudothecia develop on senescent stubble and mature during the cold (<10°C) moist conditions of autumn and winter when crops are sown and are becoming established in southern Australia (Galloway and MacLeod, 2002; Tivoli and Banniza, 2007). Initial infection occurs *via* ascospores released in the winter months that can spread to a distance of 400 m by wind and neighboring plants by rain splash (Galloway and MacLeod, 2002). Secondary infection occurs *via* rain-splashed conidia that can spread several meters within the crop (Galloway and MacLeod, 2002). Seed-borne inoculum can also introduce the pathogen to new growing regions and will establish disease in the new crop by infection of emerging seedlings (Kaiser, 1997). Optimal conditions for disease development are cool temperatures (10–15°C) with high humidity where continued leaf wetness and a high inoculum load can rapidly promote disease early in the season (Kohpina et al., 2000; Tivoli and Banniza, 2007; Hawthorne et al., 2012a,b).

The most effective and sustainable means of control is through the development of resistant cultivars (Kaur et al., 2014). Worldwide, resistance to AB has been reported to be under the control of both single major genes and polygenic modes of inheritance (Hanounik and Robertson, 1989; Rashid et al., 1991; Kohpina et al., 2000; Roman et al., 2003; Avila et al., 2004; Sillero et al., 2010). Environmental conditions can influence and complicate disease assessment (Kharbanda and Bernier, 1980; Zakrzewska, 1988; Hanounik and Robertson, 1989; Roman et al., 2003). In Australia, AB disease scores from naturally infected faba bean field trials are closely correlated with artificially inoculated shade house tests (Kimber et al., 2013). This gives assurance that results from shade house phenotyping of isolates can be reliably extended to expectations of field disease levels and can be useful for screening in the breeding program.

The southern node of the Australian Faba Bean Breeding Program (formerly managed by Pulse Breeding Australia) is based at the University of Adelaide in SA. In the southern region, which includes SA and VIC, the faba bean cultivars with resistance to AB include PBA Samira and PBA Zahra, released in 2013 and 2015, respectively (Paull, 2014, 2016). Nura, released in 2004, is also an AB-resistant cultivar and shares a potential source of resistance from Ascot with PBA Samira (Paull, 2006). Farah, released in 2001, was resistant to AB at that time (Paull, 2003, 2004) and was the dominant cultivar before the release of PBA Samira (*J. Paull, pers. comm.* 2022). PBA Bendoc and PBA Amberley, released in 2018 and 2019, are also AB-resistant cultivars (Paull, 2017; PBA Amberley, 2019).

To avoid losing AB resistance, the inclusion of multiple resistance genes or sources of resistance in a breeding program should be complemented by using different pathogen isolates during screening and resistant plant selection (Bond et al., 1994; Kohpina et al., 1999; Avila et al., 2004). Physiological specialization between faba bean genotypes and *A. fabae* isolates has been reported (Kharbanda and Bernier, 1980; Hanounik and Robertson, 1989; Rashid et al., 1991; Kohpina et al., 1999) and resistance on leaf and stem has been suggested to be under different modes of genetic control (Rashid et al., 1991; Kohpina et al., 1999; Avila et al., 2004). However, despite *A. fabae* being a sexually reproducing pathogen, there was little or no evidence for pathogenic variation in Australian *A. fabae* populations before 2013 (Kohpina et al., 1999; Kimber et al., 2009).

Farah remained resistant to AB for over a decade until the discovery of isolates in 2013 that were able to overcome its resistance (Kimber et al., 2013). A change in the AB reaction was reported in a commercial crop of Farah in northern NSW and on previously resistant faba bean lines in a breeding trial in the Lower North of SA (Kimber et al., 2013). Shade house-testing of isolates collected from these sites revealed that they were able to infect Farah, as well as PBA Rana, which had been resistant to AB at the time of its release in 2011, along with several breeding lines (Kimber et al., 2013). This indicated that at least two resistance genes may have been compromised and was considered to be evidence that a new isolate group of *A. fabae*, then named pathotype 2 in industry publications, had emerged in the pathogen population (Kimber et al., 2013). In our study, these isolates are called pathogenicity group (PG) PG-2, and the older isolates that are not virulent on Farah are named PG-1. PGs describe the host specificity of isolates based on a defined set of host differential germplasm, without inferring a specific molecular mechanism of host-pathogen interaction that normally applies to pathotypes or races (Van Der Does and Rep, 2007).

In 2014, a large-scale glasshouse screening trial was conducted on breeding material and previously selected AB-resistant germplasm accessions. This identified that most lines were susceptible to the new isolates (Kimber et al., 2015). In 2015, 38 isolates collected during 2014 and 2015, mostly from the Mid North of SA, were tested in controlled environment conditions and 71% were virulent on PBA Rana and Farah (Davidson et al., 2016a). A test of historical isolates collected from the Mid North from 1991 to 2013 revealed that the new isolate group was not present before 2012 (Davidson et al., 2016a); however, one isolate from 2012 could overcome the resistance in PBA Rana and PBA Zahra (Davidson et al., 2016a; Kimber et al., 2016). Subsequent testing of isolates collected from 2012 to 2015 identified a virulence pattern similar to the 2012 isolate, in several isolates collected in SA and VIC and indicated the emergence of distinct PGs and the potential for loss of resistance in Farah, PBA Rana, and PBA Zahra (Kimber et al., 2016).

This study reports on the phenotypic variability of the *A. fabae* population in southern Australia on commonly grown faba bean cultivars and investigates the genetic diversity of *A. fabae* isolates held in the South Australian Research and Development Institute (SARDI) pulse pathology isolate collection. This study aimed to determine (1) the range of aggressiveness levels among recent Australian isolates of *A. fabae* collected over five seasons against a faba bean differential set in ambient conditions with irrigation, (2) the number of PGs present in the pathogen population, and (3) the genetic diversity of the pathogen population.

## Materials and Methods

### Isolate Collection From Field Trials and Commercial Crops

Faba bean plants showing typical AB lesions on leaves, stems, and pods were collected from commercial crops and field trials in SA, VIC, and NSW from August to October 2015 to 2018. Seeds affected by AB lesions were collected in December during harvest. Diseased plant material was also received from agronomists and growers as diagnostic samples throughout the growing season. The number of leaves, stems, pods, or seeds sampled varied and was dependent on the presence of lesions with AB pycnidia. Host cultivar, location of the nearest town, and date collected were recorded for each sample. To isolate causal fungi, small sections of diseased leaf or stem were surface-sterilized by immersion in 70% ethanol (30 s), and 1% sodium hypochlorite (30 s), rinsed in sterile water and dried on sterile filter paper in a laminar flow cabinet. Seeds were surface-sterilized using the same method but with 2 min in 1% sodium hypochlorite. Leaf and stem material were aseptically sliced into thin pieces (approximately 10 mm by 4 mm) with a No. 22 scalpel and placed onto potato dextrose agar (PDA) (Oxoid^®^), amended with 0.01% streptomycin. Plates were incubated for approximately 14 days under fluorescent lights (two Phillips TLD 36W/840 daylight tubes and one NEC black fluorescent light), with a 12-h photoperiod at 24°C. The resulting isolates were identified as *A. fabae* based on conidial morphology according to Jellis and Punithalingam (1991) and sub-cultured to fresh PDA, from which single conidium-derived isolates were prepared and stored in sterile water at 4°C.

### Isolate Collection From Infested Faba Bean Stubble

Isolates were also sourced *via* AB-infested Farah faba bean stubble collected at harvest from the 2015 breeding trial at Saddleworth in the Mid-North of SA following the protocol used by Davidson et al. (2016b). Briefly, pots of faba bean seedlings were placed in immediate proximity to the infested stubble in ambient conditions, leading to infection from conidial and ascospore spread. Pots were sown to the faba bean cultivars Icarus (susceptible reference), PBA Rana, PBA Zahra, PBA Samira, Farah AR, and Nura AR. Farah AR and Nura AR are pure “ascochyta-resistant” lines made within the breeding program, each of which was generated by three generations of self-pollination with a single plant selection for resistance to an inoculum of mixed PG-1 *A. fabae* isolates at each generation (*J. Paull, pers. comm.* 2019). AR lines are presumed to be genetically stable for their source of ascochyta resistance and known not to be outcrossed. The breeding program retains pure (“ascochyta resistant” = AR) lines for crossing and genetic studies as faba beans are open-pollinated. Seeds from open-pollinated plants can have mixed genetic material leading to genetic drift, and impure lines may have higher than expected levels of disease than the commercial cultivar and lead to the presumption that there has been a change in the resistance of that cultivar. Seedlings were watered as required and observed for lesion development. Single conidium-derived isolates were collected from the resulting faba bean lesions and stored as described above.

### Phenotyping Isolates on Differential Host Set

For each collection year from 2015 to 2018, 40 single conidium-derived isolates were chosen as representatives from the range of cultivars and locations from the total pool of isolates collected in that season. These were tested on a host differential set of faba bean cultivars in a series of experiments in a shade house at the Waite Campus, Urrbrae, SA (34°58′15.6″S 138°38′19.4″E). The host differential set was chosen to represent the different sources of AB resistance in the breeding program and the commercial cultivars commonly grown in southern Australia. This included the susceptible control cultivar, Icarus, and cultivars with varying levels of AB resistance, Farah AR, PBA Rana, PBA Zahra, PBA Samira, and Nura AR. A full summary of the properties of the differential set is provided in Table 1. Due to seed supply considerations, Farah AR was replaced with Farah for testing of isolates collected in 2016, and PBA Samira was replaced by Samira AR (the pure selection of PBA Samira) for testing of isolates collected in 2017 and 2018. Samira AR was generated within the breeding program through three generations of self-pollination with a single plant selection for resistance to Farah-virulent isolate 59/13 at each generation (*J. Paull, pers. comm.* 2019). Pure selections of PBA Rana and PBA Zahra were not used because their resistance had already been reported to be partially compromised (Davidson et al., 2016a; Kimber et al., 2016) and their sources of resistance were no longer of interest to the breeding program. Each annual test of 40 isolates was conducted as 2 separate experiments, each with 20 isolates for a total of 8 separate experiments. The experimental design was four-replicate split-plot blocks with isolates as the main plots and faba bean cultivars as subplots randomly allocated within main plots. Two reference isolates, isolate 88/10 representing PG-1 and isolate 59/13 representing PG-2 (Kaur et al., 2014), were included in each experiment for comparison of isolate responses between the experiments. Faba bean seeds of differential cultivars were sown separately into 2.1-L round pots (three seeds per pot) filled with Van Schaik’s Biogro (Biogro Pty Ltd.) pine bark potting mix. The pots were placed on benches in an irrigated shade house in May in ambient winter conditions. Seedlings were watered as required.

**TABLE 1:** Faba bean host differential cultivars or selections used in annual pathogenicity testing and the justification for their inclusion.

Host	Justification for inclusion in the differential set
Icarus	A susceptible cultivar.
Farah OR Farah AR^a^	Farah, lost effective AB resistance in 2013 when a new isolate reaction group, PG-2, of AB was identified (Kimber et al., 2013).
PBA Rana	Popular southern Australian commercial cultivar with similar reaction as PBA Zahra to both PG-1 and PG-2 of AB but with a different pedigree to PBA Zahra^b^.
PBA Zahra	Popular southern Australian commercial cultivar with similar reaction as PBA Rana to both PG-1 and PG-2 but with a different pedigree to PBA Rana^b^.
PBA Samira OR Samira AR^a^	A commercial cultivar was released in 2013 with a high level of resistance to AB including PG-2. The source of resistance is presumed different from that in Nura^b^.
Nura AR^a^	Nura was released in 2004 with a high level of resistance to AB including PG-2 and a different pedigree from the other commercial cultivars in the differential set. The source of resistance is presumed different from that in PBA Samira^b^.

*PG, pathogenicity group.*

*^a^AR lines are “ascochyta resistant” selections of the commercial cultivars which were generated within the breeding program. These pure selections are not open-pollinated and are presumed to be pure and stable for their source of resistance.*

*^b^J. Paull, pers. comm. 2019.*

The stored single conidium-derived isolates were grown on PDA for 14 days and a conidial suspension of each isolate was produced by scraping the culture surface with a No. 22 scalpel to transfer conidia to a 10-mL tube containing sterile water. Tubes were agitated to disperse conidia and 150 μL was spread across each of four fresh PDA plates to produce sufficient cultures for inoculation. These spore-spread cultures were grown for 21 days as described above. A conidial suspension (150 mL) of each isolate was prepared by flooding the plates with tap water and gently rubbing the culture surface with a No. 22 scalpel to suspend the conidia. Spore concentrations were determined by hemocytometer, adjusted to 1 × 10^6^ conidia per mL, and surfactant Tween 20 (0.01%) (Merck Pty Ltd.) was added. Seedlings were inoculated with *A. fabae* isolates at the 3–4 node growth stage. Each conidial suspension was sprayed separately until runoff onto four replicate pots of each faba bean cultivar when plants were approximately 5 weeks old. Control plants (four pots per faba bean cultivar) were sprayed with tap water plus Tween 20 (0.01%) until runoff.

After inoculation, faba bean plants received overhead misting (Eindor nozzles 700 180 LPH Plas-Flow Irrigation), for 30 s every 30 min each day to maintain leaf wetness until disease assessment. The disease was assessed on each plant once typical AB lesions were well-established, ranging from 4 to 7 weeks post-inoculation. Assessments incorporated the percent area of leaf lesions and the percent area of stem lesions of the nodes and internodes that had been inoculated per plant. Leaf severity and stem severity were averaged to calculate the percent of plant disease and square root transformed to satisfy assumptions of normality and normalize residuals where necessary. Each experiment of 20 isolates plus reference isolates was analyzed separately using a split-plot analysis of variance with isolate as the main plot in GenStat^®^ version 17. Cultivar by isolate reactions were placed into one of four categories: Resistant (R), Moderately Resistant (MR), Moderately Susceptible (MS), or Susceptible (S) using the least significant difference (LSD) between mean disease scores based on 95% confidence intervals. Isolates that caused a Resistant (R) reaction were the least pathogenic, in the lowest LSD category, and caused less than 2% of whole plant diseases.

### Isolate Pathogenicity Group Designation

Isolates were grouped according to the severity of disease on Farah/AR, PBA Samira/Samira AR, and Nura AR as these represent the major types of AB resistance present in the breeding program (*J. Paull, pers. comm.* 2019). PG designations for *A. fabae* isolates are as specified in Table 2.

**TABLE 2:** Pathogenicity group assignment for *Ascochyta fabae* reactions on differential faba bean cultivars.

	Pathogenicity group		
	1	2	3^a^
Farah/AR	R	MR to S	MR to S
PBA Samira	R	MR	MS to S
Samira AR	R	R	MR to S
Nura AR	R	R	MR to S

*Reactions are R, resistant; MR, moderately resistant; MS, moderately susceptible; S, susceptible. ^a^A third pathogenicity group was identified based on grouping cultivar reactions as shown.*

### Molecular Identification of Mating Type (MAT1–1 and MAT1–2)

Single spore isolates were grown on PDA as described above. DNA was extracted from mycelia using the SARDI Molecular Diagnostics DNA extraction service (Ophel-Keller et al., 2008), quantified using Quant-iT™ Picogreen^®^ dsDNA reagents and kits (Invitrogen, Thermo Fisher Scientific, Waltham, MA, United States), and diluted to 1 ng. μL^–1^ before being used in PCR. Mating types were identified using MAT1–1-specific forward primer (5′-GCAACATCCTAGCATGATG-3′), MAT1–2-specific forward primer (5′-CTGTCTCACCCAAGGCAAAC-3′), and common reverse primer (5′-CACATCACCCCACAAGTCAG-3′) in a multiplex PCR (Chérif et al., 2006). PCR was carried out using 4 μL of 1 ng. μL^–1^ DNA in a 20 μL total volume and cycling conditions were as follows: 95°C for 15 min; 40 cycles of 95°C for 30 s, 60°C for 30 s, 72°C for 40 s; and 72°C for 10 min. Amplified fragments were diluted at 1:10 and resolved on 2% agarose gel. The expected amplicon sizes were 450 and 750 bp for mating types MAT1–1 and MAT1–2, respectively. Reference isolates for each mating type and negative controls were included in each set of PCR reactions.

### DNA Extraction and DArTseq Genotyping

DNA for DArTseq was extracted from liquid culture *A. fabae* mycelium for 94 isolates using a modified CTAB DNA isolation method (Xin and Chen, 2012). Isolates were grown in an 80-mL yeast extract glucose liquid medium in 250 mL flasks with shaking at 180 rpm at 22°C. Mycelia was washed with sterile water in a muslin cloth and freeze-dried for later grinding and DNA extraction. DNA samples were diluted in water to a concentration of 50–100 ng.μL^–1^. For a further 213 *A. fabae* isolates, genomic DNA was extracted from mycelium collected from PDA plates, using an in-house phenol-chloroform-isoamyl alcohol-based extraction method, which was optimized for yield and quality. Clean DNA was diluted in Tris-EDTA buffer containing 40 μg.mL^–1^ RNase A. DNA concentration was determined using a NanoDrop^®^ Spectrophotometer (Thermo Fisher Scientific). DNA quality and purity were assessed by agarose gel electrophoresis.

The DArTseq genotyping was performed by Diversity Arrays Technology (Bruce, ACT, Australia), which yields single nucleotide polymorphism (SNP) and presence/absence variant markers, designated as SilicoDArT markers by Diversity Arrays. Both marker types were processed in Excel to filter and remove loci with greater than 20% missing values. Loci were also removed from further analysis if minor allele frequency was less than 0.02.

### Population Genetics Analysis

Population structure was determined using the program, STRUCTURE v2.3.4 (Pritchard et al., 2000; Evanno et al., 2005) under the admixture model, and the optimum number of clusters, K was determined using the Structure Harvester web server at http://taylor0.biology.ucla.edu/structureHarvester/ (Earl and Von Holdt, 2012). Initial Markov Chain Monte Carlo (MCMC) runs consisted of 2,000 burn-in replicates and 20,000 MCMC replicates, for values of K-inferred clusters from 1 to 6 (SilicoDArT markers) or from 1 to 8 (SNP markers). Three replicates of each run were generated. The final population structure was constructed with 10,000 burn-in replicates and 50,000 MCMC replicates with K-inferred clusters set at an optimum number of 3 for both SilcoDArT and SNP markers.

Filtered SNP and SilicoDArT marker sets for *A. fabae* were prepared in simple data frame format with the first column containing the sample names and the first row with the locus names. Population genetics analyses were implemented in the open-source statistical platform R (version 4.0.5; R Core Team, 2021) using the package poppr version 2.8.7 (Kamvar et al., 2014, 2015) with key dependencies, ade4 (version 1.7-16), and adegenet (version 2.1.3). Hierarchical clustering of isolates was performed with the ‘‘aboot’’ function in poppr to produce Unweighted Pair Group Method Arithmetic mean (UPGMA) trees for all isolates using SNP or SilicoDArT genotype data. The distance was defined as Nei’s genetic distance, and 10,000 bootstrap replications were performed. Trees were drawn using the Interactive Tree of Life (ITOL) webserver (version 6^1^).

Pairwise fixation indices (F_st_) between populations were calculated using the genet.dist WC84 method in the R package hierfstat (Yang, 1998; Goudet, 2005) for F_st_ calculation by the method of Weir and Cockerham (1984). Isolates were classified by the available collection metadata into populations including the year of collection (2014, 2015, 2016, 2017, and 2018), collection region [Yorke Peninsula (YP), Upper North (UN), Mid-North (MN), Lower-North (LN), South East (SE), Victoria (VIC)], mating type (MAT1–1, MAT1–2), PG (1, 2, 3), and host variety (Fiesta, Farah, Farah AR, PBA Rana, Nura, Nura AR, PBA Samira, PBA Zahra). Multilocus genotypes (MLGs) and associated population genetics statistics, Shannon–Weiner diversity index H (Shannon, 1948), and Nei’s unbiased gene diversity H_exp_ (Nei, 1978), were calculated for original MLGs. Wright’s Fixation Indices were calculated using the Weir and Cockerham (1984) method.

### Genome Sequencing and Analysis

Illumina sequencing of three *A. fabae* isolates, 206/15, FT15036, and 247/15, was performed by sequencing Nextera (Illumina, San Diego, CA, United States) genomic libraries on an Illumina NextSeq instrument. Isolate 206/15 caused an MR reaction, and isolate FT15036 caused an R reaction, on the pure selection Samira AR and were classified as PG-2. Isolate 247/15 caused an MS reaction on commercial cultivar PBA Samira and an MR reaction on the pure selection Nura AR and is classified as belonging to PG-3. Genomic DNA was prepared from liquid-culture mycelium samples for each isolate using the CTAB method described above for DArTseq genotyping. Sequencing libraries were prepared with 50 ng of genomic DNA for each sample using the Nextera DNA library preparation kit and the compatible Nextera index kit (Illumina) according to the manufacturer’s instructions. Multiplexed libraries were loaded onto the Illumina NextSeq 500 sequencer and 150 bp paired-end reads were acquired and saved to the BaseSpace archive (Illumina). Genome sequencing quality for raw Illumina data was assessed using FastQC 0.11.52 and indexed reads were trimmed using Trimmomatic 0.38 (Bolger et al., 2014). Contigs were assembled using SPAdes version 3.11.1 (-k 21,33,55,77,99,127 –careful) (Bankevich et al., 2012). Sequencing statistics were calculated from the draft assemblies for each of the isolates excluding contigs shorter than 500 bp, using Quast version 4.6.2 (Gurevich et al., 2013). *A. fabae* genome assemblies (BioProject PRJNA506513, and SRA SRX5096512, SRX5096511, and SRX5096508) were subsequently used in the current study to confirm the distribution of markers and to map trait-associated loci to the assemblies for candidate gene prediction. Gene prediction was completed with GeneMark-ES version 4.33 (options: –ES–fungus) (Lomsadze et al., 2005; Ter-Hovhannisyan et al., 2008). The *A. fabae* genome sequencing data and assemblies are archived at NCBI BioProject PRJNA506513. To improve Genome-Wide Association Study (GWAS) trait association prediction, RagTag version 1.0.1 (Alonge et al., 2019) was used to complete correction and scaffolding steps for Illumina *A. fabae* assemblies, with the published PacBio genome assembly for *Ascochyta lentis* isolate Al4 (GCA_004011705.1; Lee et al., 2021) as the reference sequence. GWAS was performed using the mapped positions to original Illumina contigs and RagTag scaffolds for each isolate aligned to the near-complete *A. lentis* assembly. Circos plots to show homology between *A. lentis* Al4 and *A. fabae* RagTag assemblies, and SNP and SilicoDArT marker positions on the RagTag assemblies, were generated in Circos version 0.69-8 (Krzywinski et al., 2009). Homology between *A. lentis* and *A. fabae* sequences was determined using nucmer (Kurtz et al., 2004) version 4.0.0 with the “–mum” argument to return unique regions with sequence identity above 70%. Percent GC content was calculated for 10-kb windows using the “bedtools makewindows,” “bedtools getfasta,” and “bedtools nuc” commands in Bedtools version 2.26.0 (Quinlan and Hall, 2010).

### Genome-Wide Association Study

A GWAS analysis of *A. fabae* isolates, for which disease phenotypes and DArTseq genotypes were available, was performed using the R package, Genome Association, and Prediction Integrated Tool (GAPIT version 3; Lipka et al., 2012; Tang et al., 2016). Trait files were derived from disease phenotyping scores normalized to the mean disease score for the susceptible faba bean control cultivar Icarus in each independent phenotyping experiment. Quantitative traits designated for differential cultivars were Farah AR, Farah, Rana, Zahra, Samira AR, Samira, Nura AR, and the PG. Genotype files were formatted in HapMap format in which marker name, mapped contig and position, and genotype for each isolate are contained. Marker positions were determined using BLASTn searches (Altschul et al., 1990) against *A. fabae* genome assemblies for isolates 247/15, 206/15, and FT15036 scaffolded using RagTag and nucmer as described above. The mixed linear model (MLM), multiple-locus mixed linear model (MLMM), and fixed and random model circulating probability unification (FarmCPU) (Liu et al., 2016) models were tested in GAPIT. Trait associations above the significance threshold calculated for each data set using the Bonferroni *p*-value correction method were designated as significant trait-associated loci. Genomic regions of interest were extracted from RagTag scaffolds, to which the respective markers were mapped, and proteins annotated on matching contigs from the original assemblies were tested for the presence of signal peptide and effector likelihood using the web server-based programs signalP version 5.0 (Almagro Armenteros et al., 2019) and effectorP version 3.0 (Sperschneider and Dodds, 2021).

## Results

### Isolate Collection

**FIGURE 1:**
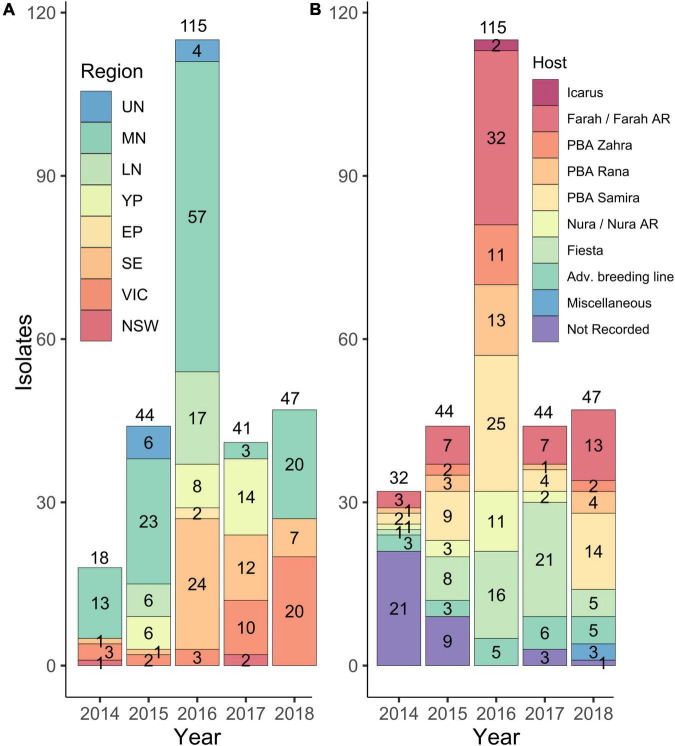
Numbers of *A. fabae* isolates were collected from 2014 to 2018 from South Australia (SA), Victoria (VIC), and New South Wales (NSW) and held in the South Australian Research and Development Institute (SARDI) isolate collection. Isolates are grouped by region **(A)** and by host cultivar from which they were collected **(B)**. Regions are abbreviated as follows: Upper North SA, UN; Mid North SA, MN; Lower North SA, LN; Yorke Peninsula SA, YP; Eyre Peninsula SA, EP; South East SA, SE; Victoria, VIC, and New South Wales, NSW. Region classifications in **(A)** do not include isolates collected in 2014 with no region recorded (14) and do not include those collected in 2017 from Adelaide City (2) and Fleurieu (1). Host cultivar classifications in **(B)** included some advanced breeding lines (Adv. breeding line). In 2018 three isolates classified as Miscellaneous, were collected from cultivars Aquadulce, Doza, and Fiord, respectively.

A total of 318 isolates were collected from AB lesions on faba beans from 1991 to 2018 from SA, VIC, and NSW, and mating types were identified using mating type-specific PCR markers. Of these, seven isolates were identified not to be *A. fabae* and were excluded from further analysis. Of the remaining 311, only 29 isolates (9.3%) were collected from 1991 to 2013 before the previously resistant Farah became susceptible to AB (Kimber et al., 2013) and when targeted monitoring of *A. fabae* was not undertaken. In 2014, there was increased collection activity by the SARDI pulse pathology program and of the aforementioned 311 *A. fabae* isolates, 32 (10.3%) were collected in 2014, 44 (14.1%) in 2015, 115 (37.0%) in 2016, 44 (14.1%) in 2017, and 47 (15.1%) in 2018. Collection tallies for the years 2014–2018 are summarized in Figure 1 and are grouped by region (Figure 1A) and by host cultivar (Figure 1B).

### Phenotyping Isolates Collected From 2015 to 2018

**FIGURE 2:**
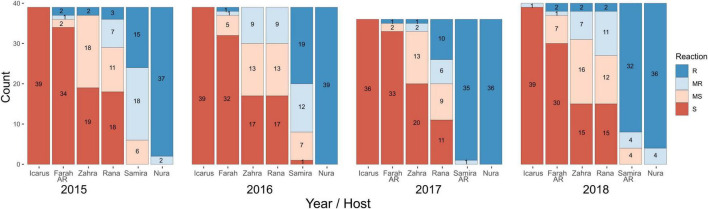
Isolate pathotyping using differential faba bean cultivars. Isolates collected from 2015 to 2018 were screened for disease response using a differential faba bean set that included cultivars: Icarus (susceptible control), Farah AR (2015, 2017, and 2018), Farah (2016), PBA Zahra, PBA Rana, PBA Samira (2015 and 2016), Samira AR (2017 and 2018), and Nura AR. Isolates (*n* = 36–40 each year) were classified as resistant (R), moderately resistant (MR), moderately susceptible (MS), or susceptible (S) according to LSD for the respective experiment in which the isolates were tested. Stacked bars show isolate counts for each reaction type in differential cultivars.

*Ascochyta fabae* isolates collected in the growing seasons of 2015 through to 2018 inclusive were inoculated onto the faba bean differential cultivars (Table 1) to determine their disease responses. Of the isolates tested, approximately two-thirds were collected from field trials and one-third from commercial crops. Plant disease percent severity on the susceptible cultivar Icarus ranged from 56 to 100% across the experiments conducted over 4 years. The LSD statistic for square root-transformed plant disease percent severity was used to determine each of the disease categories within each test and ranged from 0.90 to 1.234 across the eight experiments, 20 isolates per experiment for annual testing of 40 isolates. Results are shown in Figure 2 with stacked bar plots for the six differential cultivars, grouped for each collection year. On the most susceptible cultivar Icarus, almost all isolates were highly aggressive, while on the most resistant Nura AR, almost all isolates caused a resistant (R) response with only a small number causing a MR response. The remaining cultivars showed a range of reactions to isolates from R through to S. Most isolates caused an MS or S reaction on Farah/Farah AR and an R or MR reaction on PBA Samira/Samira AR.

**FIGURE 3:**
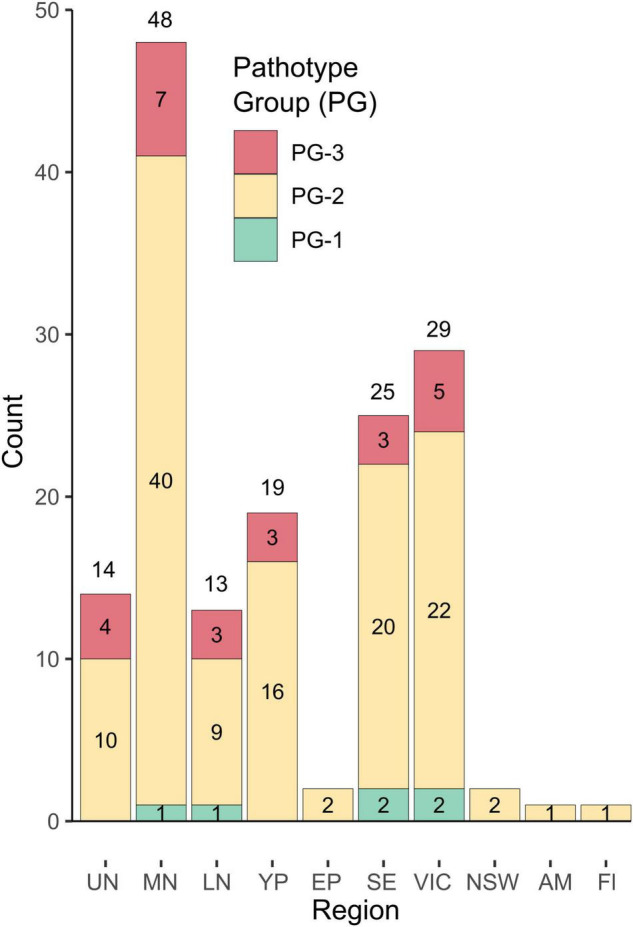
Distribution of pathogenicity groups in geographic regions for isolates collected from 2015 to 2018. Isolates were classified as pathogenicity group (PG), PG-1, PG-2, or PG-3 based on the differential host cultivar reaction. Region abbreviations are as follows: Upper North SA, UN; Mid North SA, MN; Lower North SA, LN; Yorke Peninsula SA, YP; Eyre Peninsula SA, EP; South-East SA, SE; Victoria, VIC, and New South Wales, NSW, and single isolates from an Adelaide Metropolitan site (AM) and a location on the Fleurieu Peninsula (Fl) south of Adelaide were also included.

Pathogenicity groups were designated for all isolates that were phenotyped in differential disease assays, with PG-1 isolates being aggressive only on Icarus, and PG-2 isolates being aggressive on Icarus, as well as Farah/Farah AR, PBA Zahra, and PBA Rana but not PBA Samira or Nura AR. A third PG classification, PG-3, comprised isolates also being aggressive on PBA Samira, Samira AR, or Nura AR. Figure 3 shows the PGs of 154 isolates collected from 2015 to 2018 based on their differential disease response phenotypes. PG-2 isolates were detected in every region in each year of collection and accounted for 80% of the total isolates collected. Six isolates (4%) were classified as PG-1. Isolates with the PG-3 classification were also detected each year and were found in all regions across the 4 years of testing, except in NSW, Eyre Peninsula, Adelaide Metro, and Fleurieu Peninsula. PG-3 accounted for 16% of the total isolates collected.

### Mating Type Structure of *Ascochyta fabae*

Mating types of 311 isolates were determined using mating type-specific PCR. To statistically test the hypothesis that mating type ratios were not different from 50:50, we applied the Chi-square (χ^2^) test. Results revealed equal ratios of MAT1–1 and MAT1–2 mating types in the pathogen population for isolates grouped by year, region of collection, or host cultivar (Supplementary Table 1). An additional assessment of mating types for isolates grouped by collection year was made for PG-2 isolates because this was the largest group of isolates (123 isolates) within the 154 isolates phenotyped for disease response. In all year, region, and host cultivar categories, and for collection year for the PG-2 isolates, the χ^2^ test revealed an equal ratio of MAT1–1 and MAT1–2 mating types. There were 286 isolates collected from the major faba bean growing regions (Lower, Mid and Upper North, Yorke Peninsula, Southeast, and VIC), and equal ratios of MAT1–1 and MAT1–2 were revealed in each of these major regions for isolates collected over the entire collection period. The number of isolates in the remaining regions (Eyre Peninsula, Adelaide Metropolitan, and Fleurieu Peninsula) was too small to test with χ^2^ analysis but ratios were similar to the main growing regions.

### DArTseq Genotyping Reveals a Panmictic *Ascochyta fabae* Population in Australia

A total of 305 *A. fabae* isolates from the SARDI collection were genotyped by Diversity Arrays Technology using the DArTseq method (Elshire et al., 2011; Kilian et al., 2012).

After removing markers with missing data for more than 20% of isolates and non-polymorphic markers with minor allele frequency less than 0.02, there were 751 SNP markers and 2,013 SilicoDArT markers. Filtered SNP and SilicoDArT marker data are provided in Supplementary File 1.

**FIGURE 4:**
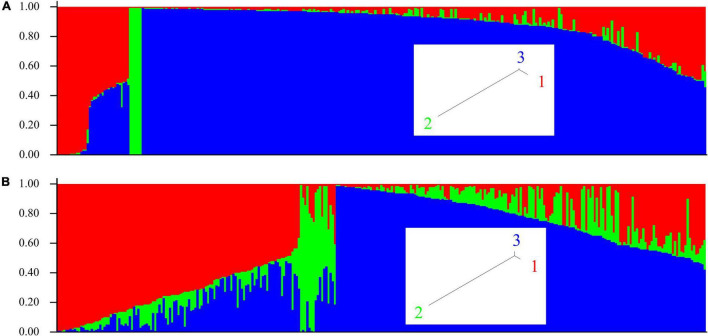
Population structure of *A. fabae* isolates collected in Australia using the program STRUCTURE. **(A)** SNP and **(B)** SilicoDArT genotype data. Inset, tree view from STRUCTURE for SNP and SilicoDArT genotype-based clusters.

Population structure for all 305 isolates in the study was determined. Using the STRUCTURE program in conjunction with Structure Harvester, we predicted three genotype-based clusters, and these were different for the two genotype datasets for SNP and SilicoDArT markers. Figure 4A shows SNP marker-based and Figure 4B shows presence/absence variant marker-based population structure, respectively. Membership of isolates in clusters determined using the two genotyping methods was not correlated (*r*^2^ = 0.001). These observations suggest that the two marker types are inherited by different mechanisms. UPGMA (Unweighted Pair Group Method with Arithmetic Mean) trees for both marker types are provided as linear dendrograms in Supplementary Figure 1.

### Population Genetics Analysis

Population-based classifications of *A. fabae* isolates were made using the available collection data categories, including collection year, region, mating type, PG, and host variety. Supplementary Table 2A summarizes the *A. fabae* population genetics statistics, including the number of individuals in each population for the different categories, which was equal to the number of multilocus groups (MLG), the Shannon–Weiner diversity index H (Shannon, 1948), and Nei’s unbiased gene diversity H_exp_ (Nei, 1978). For all population classifications, the Shannon–Weiner diversity index (H) was the same regardless of the marker type. Nei’s genetic diversity (H_exp_) was variable between marker types, with SilicoDArT markers having slightly higher genetic diversity than SNP markers. All the population classifications were shown to have similarly high levels of genetic diversity with none of the particular categories having a genetic diversity substantially different from the average value for SNP and SilicoDArT markers of 0.25 (standard deviation 0.006) and 0.34 (standard deviation 0.004), respectively. Genotype diversity was almost identical for each mating classification. It is evident that mating is unrestricted across all categories and that this feature of the *A. fabae* populations in all categories is the source of high genotype diversity.

Wright’s fixation index or pairwise F_st_ values were calculated for SNP and SilicoDArT markers and are presented in Supplementary Table 2B. These results show that populations do not bias toward particular genotypes and that there is no genetic drift over time, no selection on different host varieties or in different regions. The evidence for the region classification suggests the wide distribution of alleles in all regions and prolific mating in the crop environment in all years across the 5 years of focused isolate collection. The lack of distinct genotypes in populations based on PG indicates that no particular genotypes have increased aggressiveness or particular host cultivar specificity.

### Genome Assembly and RagTag Scaffolding Enable the Prediction of Marker Locations

Three *A. fabae* isolates were selected for whole-genome sequencing and assembly as described above. Illumina sequencing coverage for *A. fabae* isolates was between 50× and 60× and assemblies were produced using SPAdes. Table 2 shows basic sequencing and assembly statistics for the SPAdes assemblies and compares the *A. fabae* data to the published reference assembly for the closely related *A. lentis* isolate Al4 (Lee et al., 2021). The program RagTag (formerly RaGOO) (Alonge et al., 2019), was used to construct improved *A. fabae* assemblies by orienting and scaffolding the fragmented short-read contigs into larger scaffolds aligned to the *A. lentis* reference genome. By scaffolding *A. fabae* contigs according to the closely related *A. lentis*, we assume that there is synteny between the two species. In addition to Illumina assembly statistics, Table 2 shows the results of RagTag scaffolding, and subsequent whole genome alignment using nucmer (Kurtz et al., 2004) of the *A. fabae* and *A. lentis* Al4 genome files. The assembly size for isolate 247/15 was in the expected size range of 40.5 Mb and with similar %GC content to *A. lentis* at 49.2%. However, for *A. fabae* isolates 206/15 and FT15036, assembly sizes were unexpectedly high, being more than 20% larger than expected at around 50 Mb and having low %GC content at around 46%. The larger assembly size and the very high number of contigs ranging from 73,000 to 91,000, for isolates 206/15 and FT15036, suggest that these isolates have genomes with larger amounts of repetitive and AT-rich DNA sequence. Highly AT-rich genomes are more problematic for Illumina sequencing and genome assembly from short-read data. RagTag processing of all three *A. fabae* assemblies made only a small improvement to the contig number and no real change to assembly size and %GC content (Table 3). However, the combined sizes of scaffolds in the RagTag assemblies with unique nucmer matches to the *A. lentis* genome assembly, for isolates 247/15, 206/15, and FT15036, were 36.4, 43.6, and 44.1 Mb, respectively. Non-scaffolded sequences for the *A. fabae* assemblies were mostly short AT-rich contigs having average size and %GC content of 455 bp and 35%, respectively. Alignment of the three *A. fabae* RagTag assemblies to the *A. lentis* reference assembly eliminates multiple alignment matches and reveals the extent of possible misassembly of short AT-rich reads in the SPAdes assemblies for *A. fabae*, particularly for isolates 206/15 and FT15036. Nevertheless, isolates 206/15 and FT15036 may have expanded AT-rich DNA regions.

**TABLE 3:** *Ascochyta fabae* genome sequencing. Sequencing and assembly statistics for *A. fabae* genomes and RagTag assemblies scaffolded on the *A. lentis* Al4 reference genome.

Assembly	SPAdes assembly*^c^*				RagTag assembly mapping to *A. lentis*			RagTag/nucmer hits >70% identical to *A. lentis*		
			
Statistic	Coverage	Size (Mb)	Contigs	%GC	size (Mb)	Contigs	%GC	Size (Mb)	Contigs	%GC
*A. lentis* Al4^a^	288×	42.0	28	48.4	–	–	–			
247/15^b^	51×	40.5	18,293	49.2	40.1	16,632	49.1	36.4	3,171	50.4
206/15^b^	50×	53.7	91,450	45.6	54.0	89,819	45.6	43.6	3,284	47.8
FT15036^b^	60×	53.3	72,677	45.7	53.5	71,106	45.7	44.1	2,973	47.5

*^a^PacBio reference genome assembly (Lee et al., 2021).*

*^b^Illumina sequencing of A. fabae, this paper.*

*^c^Excludes contigs <500 bp.*

**FIGURE 5:**
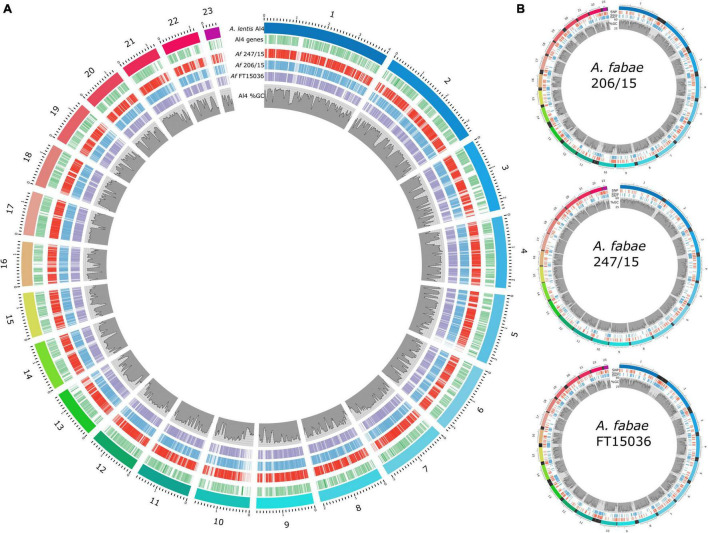
*Ascochyta fabae* genome sequencing. **(A)** Comparison of *A. fabae* genome assemblies with the published near-complete *A. lentis* assembly. Coverage of *A. fabae* RagTag genome assemblies aligned to the *A. lentis* Al4 reference genome, shown as red (247/15), blue (206/15), and purple (FT15036) blocks. *A. lentis* genes (green bars) and %GC content as previously published (Lee et al., 2021) are also shown. **(B)** Scaffolded *A. fabae* assemblies for isolates, 206/15, 247-15, and FT15036, aligned using *A. lentis* Al4. Colored bands in the outer track for each plot represent RagTag contigs aligned to *A. lentis* chromosomes 1–23 using nucmer. Mapped positions of Diversity Arrays SNP (red) and SilicoDArT (blue) loci are indicated and %GC content for respective RagTag *A. fabae* scaffolds (1–23) is plotted. Blocks of black bands in the outer tracks represent *A. fabae* contigs not included in the large RagTag scaffolds but aligned to the preceding *A. lentis* scaffold in the nucmer analysis.

Unique nucmer-aligned sequences from the RagTag assemblies are a reasonable representation of the genomes for *A. fabae* to map Diversity Arrays SNP and SilicoDArT loci. Figure 5A shows the coverage of nucmer-aligned RagTag *A. fabae* genomes along with *A. lentis*-annotated genes and %GC content (Lee et al., 2021). *A. fabae* sequence matches were distributed across the *A. lentis* genome and, consistent with the reported difficulty of sequencing and assembly through AT-rich genomic sequence using short-read Illumina technology, AT-rich regions of *A. lentis* were also regions with low sequencing coverage in *A. fabae*. It is important to note that *A. fabae* contigs excluded from the nucmer alignment were generally AT-rich and did not match any other species in BLAST searches, and we conclude that the sequencing and assembly of *A. fabae* isolates were not affected by contamination. DArTseq loci were mapped to the RagTag *A. fabae* assemblies (nucmer aligned sequence only) using BLASTn. The marker sequences and mapped positions are provided in Supplementary File 2. Figure 5B shows the positions of SNP and SilicoDArT markers mapped to the three scaffolded RagTag-scaffolded *A. fabae* assemblies. Of the combined total of 751 SNP and 2,013 SilicoDArT filtered markers (2,764 markers in total), 1,919 (69%), 1,907 (69%), and 1,334 (48%) markers were mapped to isolates 206/15, 247/15, and FT15036, respectively. Marker densities were calculated for the three isolates, for markers mapped to either GC-AT-balanced or AT-rich DNA regions. Average marker densities for SNP markers were 12 and 26 markers per Mb for GC-AT-balanced and AT-rich regions, respectively, and for SilicoDArT markers, 17 and 92 markers per Mb, respectively.

### Genome-Wide Association Study Analysis

**FIGURE 6:**
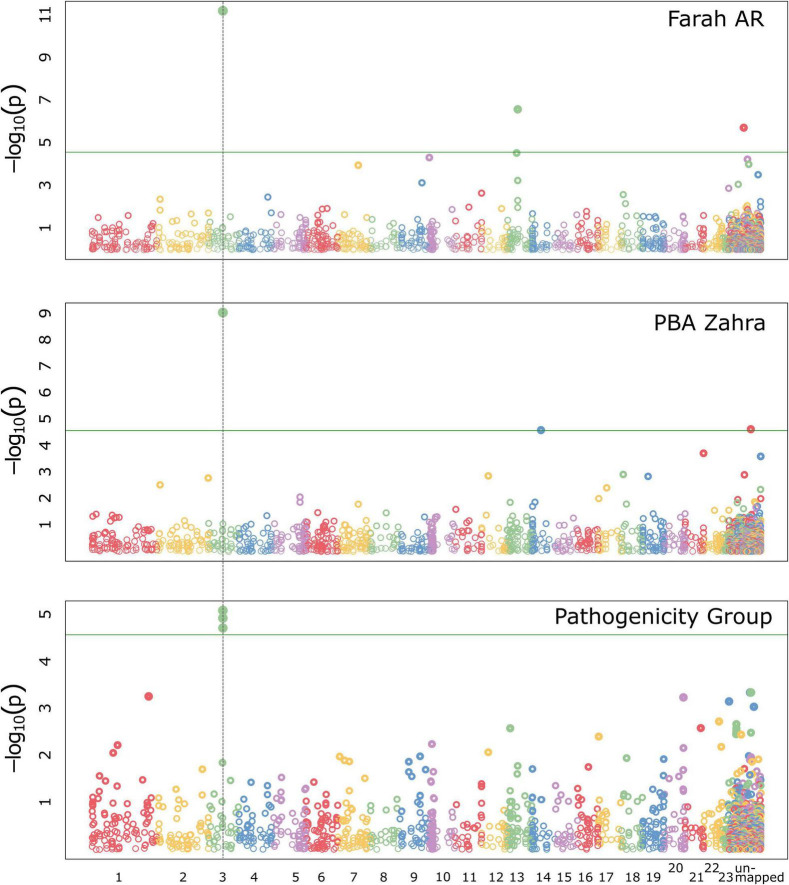
Manhattan plots for GWAS analyses implementing the FarmCPU model in GAPIT with the significant trait-associated locus (dotted vertical line) that was identified by the association of two markers mapped in *A. fabae* isolate 206/15, on RagTag scaffold 3. The locus was associated with Farah AR and PBA Zahra disease traits and for the pathogenicity group trait.

A GWAS was implemented using the program GAPIT (Lipka et al., 2012; Tang et al., 2016). Traits assessed in the GWAS included disease assay results for the faba bean differential cultivars normalized to the average result for the susceptible control, Icarus. The additional trait, PG, was given for each isolate according to the PG assignment criteria outlined in Table 2. The total number of isolates included in the GWAS was 150, however, not all isolates were tested for all traits. A detailed summary of significant trait-associated loci for each GWAS model is provided in Supplementary Table 3. Figure 6 shows Manhattan plots for the FarmCPU model, for traits where significantly associated markers on RagTag scaffold 3 were identified.

*Ascochyta fabae* 206/15-mapped markers had the strongest interaction with Farah AR and PBA Zahra, with relatively strong trait associations above the significance threshold in respective analyses. The FarmCPU model predicted trait associations for SilicoDArT markers located on the 206/15 RagTag scaffold homologous to *A. lentis* contig 3, at approximately 809 kb. In the GWAS analysis of markers mapped in 206/15 for PBA Zahra disease, the same scaffold 3 markers were significantly associated, although weakly. Additionally, a SNP marker on homologous scaffold 12 was identified as associated with PBA Zahra disease for the analysis of markers mapped in isolate 247/15 and FT15036. For Farah disease, weakly associated markers were identified for loci on homologous scaffold 1 (approximately position 2.8 Mb) and scaffold 9 (position 1.1 Mb). The PG trait identified the same markers as the Farah AR and PBA Zahra disease traits, indicating that these disease responses are key determinants.

We performed manual genome searches of the original annotated genome assemblies for isolate 206/15 to identify annotated proteins from the region associated with SilicoDArT markers Silico-33590290 and Silico-33591371 from scaffold 3. These were the most strongly associated markers with the Farah AR and PBA Zahra disease, and PG traits. Protein sequences annotated in the original *A. fabae* 206/15 assembly contigs for a 250 Mb region on RagTag scaffold 3 matching *A. lentis* Al4 chromosome 3, contained 60 protein-coding genes. Four proteins carried a secretion signal peptide and only one was predicted to be an effector using effectorP version 3. The predicted effector was present in both other *A. fabae* genome assemblies and likely does not account for phenotypic differences. A similar approach was taken for significant trait-associated loci on RagTag scaffold 13 (Farah AR; isolate 206/15), 12 (PBA Zahra; isolates 247/15 and FT15036), 1 (Farah; all isolates), and 9 (Farah; all isolates). Secreted predicted effectors were found in the vicinity of these identified regions but for each of the genes identified, there were no differences for the three isolates for which genome sequences were available. The most significant trait-associated region remains the “scaffold 3” locus that has two co-located markers (Silico-33590290 and Silico-33591371). SilicoDArT markers are presence/absence markers, and both markers are present in 206/15 but absent from 247/15 and FT15036. Of the 150 isolates in the GWAS that had both markers present (*n* = 16), the values for average normalized disease score for Farah AR and PBA Zahra disease, and PG, were 0.43, 0.36, and 1.7, respectively. For the remainder of isolates for which both markers were absent (*n* = 134), the average Farah AR and PBA Zahra disease and average PG values were 0.76, 0.55, and 2.18, respectively.

## Discussion

### Pathogenicity of *Ascochyta fabae*

This is the first report examining a comprehensive collection of *A. fabae* isolates and describing the pathogen population present in southern Australia. Variability in pathogenicity was previously reported for only six southern Australian isolates collected over 20 years ago (Lawsawadsiri, 1994; Kohpina et al., 1999). In our study, the characterization of 154 isolate phenotypes using a different set of faba bean cultivars revealed that there are three PGs, with 80% of the isolates tested being PG-2. An individual PG is defined as a group of isolates that can cause significantly different disease levels on a host cultivar with a different genetic background or presumed source of resistance. The predominance of PG-2 was previously widely reported in industry publications (Blake et al., 2019, 2020). PG-2 comprises isolates that are aggressive on Farah and cause a moderate level of infection on PBA Rana and PBA Zahra but are not pathogenic on PBA Samira, Samira AR, or Nura.

We also identified an emerging PG-3 strain in the population able to infect elite cultivars PBA Samira, Samira AR, or Nura AR at higher-than-expected levels than their current National Variety Trial AB disease rating (NVT^2^). As the population genetics analysis found no association of genotype with PG or population descriptors, identification of a new PG can currently only be revealed by disease phenotypes. PBA Samira and Nura have remained resistant to AB and are widely grown in southern Australia (Blake et al., 2017, 2019, 2020; Pearce et al., 2021). However, the presence of PG-3 isolates that can infect these cultivars is the cause of concern for the durability of the current source(s) of AB resistance.

Before 2015, only a few isolates were able to infect Farah (Kimber et al., 2016), leading to the conclusion that PG-1 was predominant. We identified that only 4% of isolates were PG-1 isolates and this indicates that a major shift in the population toward more pathogenic isolates has occurred since 2015. Thus, PG-2 and PG-3 isolates should be used to screen faba bean germplasm and elite lines for resistance within the breeding program. The use of pure “ascochyta resistant” (AR) lines will continue to be useful for testing the stability of AB resistance within the breeding program, for crossing, genetic studies, and pathogenicity testing.

McDonald and Linde (2002) described five evolutionary forces that influence pathogen populations and may contribute to the loss of effective plant resistance genes. For *A. fabae* evolution, four of these forces present a potentially high risk *viz*.: (1) large over-seasoning populations that survive on faba bean stubble maintaining virulent alleles, (2) asexual conidia are dispersed by air and the pathogen may transfer long distances on seed, (3) a mixed reproduction system that produces both annual sexual outcrossing and asexual propagules, and (4) the resistance genes are deployed in genetically uniform monocultures over large areas. The fifth force is mutation rate, but there is insufficient information in *A. fabae* to understand the contribution of this force to variability in pathogenicity in southern Australia.

Changes in the aggressiveness of pathogen populations in response to high cropping intensity and close rotations that lead to increased disease pressure and loss of cultivar resistance have been reported for AB in lentils (Davidson et al., 2016b) and chickpea (Sambasivam et al., 2020). Whilst faba bean production increased in southern Australia from 57,900 tonnes in 1991 to 327,000 tonnes in 2019 (Australian Bureau of Agricultural and Resource Economics and Sciences [ABARES], 2020), they are generally not grown in close rotation. Since 2013, faba bean growers have diversified their cultivar selections as newer cultivars became available with improved agronomic and disease resistance traits. The lack of single cultivar dominance has likely influenced the moderate level of genetic diversity across most isolates examined in this study despite sexual mating and genetic recombination occurring in the *A. fabae* population.

The spread of PG-2 most likely occurred *via* seed infection or by simultaneous selection and is unlikely to have occurred through windblown sexual ascospores as these only spread to around 100 m (Galloway and MacLeod, 2002). Seeds constitute a major source of primary inoculum for AB and the movement of infected seeds may result in new or more virulent PGs being introduced to new areas (Kaiser, 1997; Kaiser et al., 1997). In Australia, once the seed of commercial cultivars is brought onto the farm, growers tend to retain this seed on the farm from one year to the next. This practice would reduce the risk of new virulent forms being spread, as pathogen multiplication and spatial dispersal by rain splash would be limited to that farm (Tivoli and Banniza, 2007).

In this study, most of the collected and tested isolates come from field trials (70%) rather than commercial crops (30%), but all are from natural infection. Higher annual rainfall across most of SA in 2016 led to increased AB, thus, permitting higher isolate collection numbers than for other years. Nevertheless, some sampling bias may be present due to the increased focus on isolate collections for our study. A more structured sampling approach would have been preferable for statistical analysis, however, isolates can only be obtained where the disease is observed, and only isolates amenable to culturing were retained.

### Mating-Type Structure

The presence of both mating types in *A. fabae* populations has been reported in Algeria, Lebanon, Syria, the United Kingdom, Spain, and Tunisia (Kaiser, 1997; Rubiales and Trapero-Casas, 2002; Omri Benyoussef et al., 2016). In Australia, the ratio of the two mating types MAT1–1 and MAT1–2 of *A. fabae* isolates were equal, across year, region, and host cultivar. A similar study in Tunisia found this ratio differs with a two-to-one predominance of mating-type MAT1–2 in two of the four regions examined (Omri Benyoussef et al., 2016). The disequilibrium of mating types in the Tunisian *A. fabae* population suggests that sexual reproduction and randomly mating populations may not be the major influencing factor for new pathogen genotypes (Omri Benyoussef et al., 2016). Tunisia relies on imports of large quantities of faba bean seed for farmer use (Omri Benyoussef et al., 2016), and new pathogen genotypes may originate from the imported seed instead. Furthermore, the mating type of Tunisian isolates was not correlated with aggressiveness (Omri Benyoussef et al., 2016). The predominance of PG-2 isolates and equal ratio of MAT1–1:MAT1–2 in Australia indicate that there is similarly no relationship between isolate aggressiveness and mating type. However, the sample sizes of populations in each region, for both the Australian and Tunisian studies, were unequal and further study with larger, unbiased, and structured sampling is required.

### Population Genetics and *Ascochyta fabae* Genomics

Using the Diversity Arrays DArTseq genotyping approach, we have sequenced markers that are widely distributed across the *A. fabae* genome with an approximate average density of one marker every 18 kb. DArTseq genotyping has been widely used for genetic studies in plants and has recently been applied to the genetic characterization of plant pathogenic fungi with reports of the use of DArTseq markers for the chickpea AB pathogen *Ascochyta rabiei* (Bar et al., 2021), the fusarium wilt pathogen of chickpea, *Fusarium oxysporum* f. sp. *ciceris* (Sharma et al., 2014), and the causal organism of the banana black Sigatoka disease, *Pseudocercospora fijiensis* (Chong et al., 2019). DArTseq genotyping of *A. fabae* produced 751 SNP markers and 2,013 SilicoDArT markers. Analyses of population structure were performed for the two marker types independently and the Australian population can be divided into three clusters using both SNP and SilicoDArT markers. Interestingly, the membership of isolates in the three phylogenetic clusters, as determined using the program STRUCTURE, was different for the two types of markers. This implies that SNP and SilicoDArT markers are inherited through different mechanisms and possibly at different rates.

Fungal genomes, particularly those from the Dothideomycete class that comprises many plant pathogens (Ohm et al., 2012), contain gene-rich genomic regions with balanced GC-AT content interspersed with AT-rich and gene-sparse DNA sequence (Rouxel et al., 2011; Raffaele and Kamoun, 2012; Dong et al., 2015). We determined the marker density of SNP and SilicoDArT markers in both types of genomic region and found a bias for a high concentration of SilicoDArT markers in AT-rich regions. Greater variability in the presence/absence of SilicoDArT markers in AT-rich DNA regions is likely, with AT-rich regions being populated with transposable elements that mediate changes to genome sequence through loss, duplication, and rearrangement of sequence, as generally described for plant pathogens. A further mechanism that drives a higher rate of evolution in AT-rich DNA is a repeat-induced point mutation (RIP), which targets repetitive DNA (Rouxel et al., 2011). Different rates of evolution for the two types of genomic regions are reflected in the genotyping data for *A. fabae* with a higher frequency of SilicoDArT markers in AT-rich DNA regions, than SNP markers that appear to be present in GC-AT-balanced and AT-rich regions in similar proportions. This bias in SilicoDArT marker location and possibly the rate of mutation may explain the potential differences in the mechanism and rate of inheritance of the two genetic marker types.

Although three phylogenetic clusters could be established for the Australian *A. fabae* population, no pathogenicity-based phenotypic traits were associated with the genotype-based groups. Population genetics statistics showed that the Australian *A. fabae* population has a moderately high level of genetic diversity and that no haplotypes are associated with any of the isolate collection classification criteria. The genetic data for the *A. fabae* population support distribution of isolates between faba bean-growing areas *via* contaminated seed, maintenance of genetic diversity from the equal proportions of mating types, and genetic recombination through mating as a part of the seasonal life cycle of the species. The fact that there are no distinct haplotypes associated with host cultivars indicates that while Australian cultivars have been developed to have AB resistance through the targeted breeding program, haplotype is not a good indicator of overall pathogenicity or host preference of isolates. Indeed, it is more likely that new PGs or levels of increased aggressiveness are caused by mutation, loss or gain of a key virulence gene or genes or changes in the expression levels of such genes. Small changes to key genes with significant consequences for the success, or fitness, of the pathogen, could occur independently of the genome-wide markers assessed using population genetics methods.

Population genetics analyses assess the association of overall genetic structure, or haplotype, with phenotypic traits such as host specificity. In the current study, PG was used as a broad measure to capture the shift in pathogenicity in differential host cultivars with different levels of genetic resistance. In contrast, GWAS aims to capture discrete loci and tightly linked markers that associate with the host resistance or PG trait and then predict genes that may be responsible for increased aggressiveness of the pathogen or susceptibility of particular host cultivars. GWAS requires accurate mapping of genetic markers and this was accomplished by Illumina genome sequencing of three isolates and scaffolding of contigs to the *A. lentis* genome assembly (Lee et al., 2021). RagTag generated *A. fabae* scaffolds corresponding to the nearly full-length chromosome sequences for *A. lentis* based on the assumed synteny between the closely related species. While there were several markers weakly associated with Farah AR and PBA Zahra pathogenicity traits, one locus with two markers on chromosome 3 that mapped to the 809 kb position in isolate 206/15 was consistently associated with high levels of significance, for increased pathogenicity toward these cultivars, and for the PG trait. Segregation of these markers in the diversity panel and evidence of presence/absence in three genome assemblies supports natural variation in the *A. fabae* population at this locus. The genetic locus associated with these markers has the potential to be used as a predictive marker for higher aggressiveness on Farah AR and PBA Zahra. The common parentage of Farah AR and PBA Zahra provides a possible rationale for similar responses to a putative pathogen genetic factor at this locus. Further genetic studies of biparental populations that segregate for markers at the chromosome 3 locus and the respective pathogenicity traits could be undertaken to identify genes that confer differences in the Farah AR and PBA Zahra disease response.

### Implications for Breeding for Ascochyta Blight Resistance in the Faba Bean Breeding Program

To breed for AB resistance, the breeding program must screen with *A. fabae* isolates of known phenotypes that are ideally assigned to different PGs. Isolates included in pre-breeding and breeding screens must first be pathogenicity tested against a differential host set where the source of AB resistance is known and where that resistance source is different in each differential host. Genes for AB resistance in the breeding program come from widespread germplasm sources (Kimber et al., 2004, 2006, 2009). In Australia, there are several sources of resistance available for exploitation. Kimber et al. (2015) identified novel sources of resistance to PG-1 and PG-2 isolates in diverse germplasm from the Middle East, Mediterranean, Maghreb, and Northern Europe. Several QTLs have also been reported to be associated with AB resistance. In the RIL population Icarus × Ascot, three novel QTLs were reported to be associated with AB resistance to PG-2 (Kaur et al., 2014), and more recently two QTLs were identified in a Nura × Farah RIL population (Sudheesh et al., 2019). Some overlap in these QTLs may exist, for example Nura has Icarus and Ascot as parents. Specifically, Sudheesh et al. (2019) report that their loci AB_N1 and AB_N2 are comparable to Kaur et al.’s (2014) QTL-1 and QTL-4, respectively. To deploy this resistance in the Australian breeding program, pyramiding of these QTLs would ensure durable resistance to PG-2 of AB in future cultivars; however, QTLs for resistance to PG-3 should be the subject of future research.

In conclusion, our study has revealed that the *A. fabae* pathogen population in southern Australia is naturally variable in pathogenicity with a moderate level of genetic diversity. Specific isolates exist in the population that cause significant differences in disease severity on resistant faba bean hosts and some of these more pathogenic isolates are suitable for use in targeted resistance breeding. Continued isolate collections and pathogenicity testing are essential, to supply breeding programs with isolates of known pathogenicity for AB screening, and to monitor for the emergence of new PGs that may develop in the future.

## Data Availability Statement

The datasets presented in this study can be found in online repositories. The names of the repository/repositories and accession number(s) can be found in the article/Supplementary Material.

## Author Contributions

JAD, RL, and SB conceptualized the research and secured the funding. SB and MR collected, single-spored, and curated the isolates and performed the bioassays with assistance from EF and JR in 2017 and 2018. Herdina performed the mating-type PCR assays. SG, LF-C, and RL conducted the DNA extractions and DArTseq analysis. LF-C, JWD, and RS conducted the genome sequencing and assembly. RL conducted the population structure and GWAS analysis. SB and RL wrote the manuscript. All authors read and approved the final version of the manuscript.

## Conflict of Interest

The authors declare that the research was conducted in the absence of any commercial or financial relationships that could be construed as a potential conflict of interest.

## Publisher’s Note

All claims expressed in this article are solely those of the authors and do not necessarily represent those of their affiliated organizations, or those of the publisher, the editors and the reviewers. Any product that may be evaluated in this article, or claim that may be made by its manufacturer, is not guaranteed or endorsed by the publisher.

## Footnotes

1
https://itol.embl.de/

2
nvt.grdc.com.au
