# Supplementary material for: Phenotypic and Genotypic Diversity of Ascochyta fabae Populations in Southern Australia
Source: Front Plant Sci. 2022 Aug 2;13:918211. doi: 10.3389/fpls.2022.918211 (PMC9380778; doi:10.3389/fpls.2022.918211)
Supplement: Supplementary file 1 [file Data_Sheet_1.zip › Supplementary Figure 1.pdf]

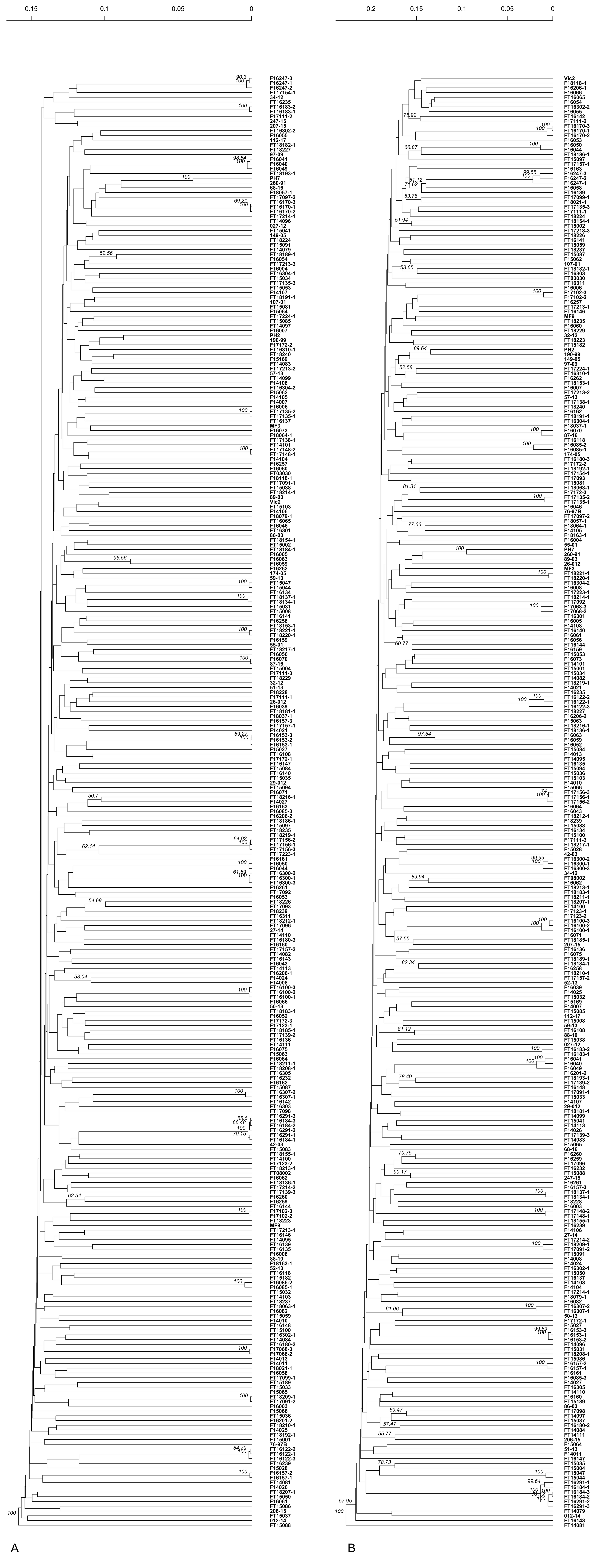

Supplementary Figure 1 UPGMA Trees for 305 *A. fabae* isolates constructed using SNP markers (A) and SilicoDArT markers (B).
