# Supplementary material for: Phenotypic and Genotypic Diversity of Ascochyta fabae Populations in Southern Australia
Source: Front Plant Sci. 2022 Aug 2;13:918211. doi: 10.3389/fpls.2022.918211 (PMC9380778; doi:10.3389/fpls.2022.918211)
Supplement: Supplementary file 1 [file Data_Sheet_1.zip › Supplementary Table 1.docx]

**Supplementary Table 1. Mating types and chi-square (χ^2^) analysis to test mating-type ratios.** Numbers of isolates, and percentage of the total number of isolates in parentheses, for each year, region, or host classification, with mating types MAT1‑1 and MAT1‑2, grouped by (A) year of collection, (B) region of collection for the major growing regions, (C) year of collection for Pathogenicity Group (PG)‑2 isolates only and (D) host cultivar. χ^2^ test statistic and corresponding *P* value for each group of isolates are provided and where *p* > 0.05 the null hypothesis of no significant difference for mating type ratio from 50:50, was supported at the 95% confidence level.

| **Grouped by:** | **Classification** | **MAT1-1** | **MAT1-2** | **Total** | **χ^2^ (*P* value )** |
| --- | --- | --- | --- | --- | --- |
| A. Year | 1991-2013 | 17 (59) | 12 (41) | 29 | 0.43 (0.510) |
|  | 2014 | 20 (63) | 12 (38) | 32 | 1.02 (0.313) |
|  | 2015 | 21 (48) | 23 (52) | 44 | 0.05 (0.831) |
|  | 2016 | 60 (52) | 55 (48) | 115 | 0.11 (0.742) |
|  | 2017 | 21 (48) | 23 (52) | 44 | 0.05 (0.831) |
|  | 2018 | 29 (62) | 18 (38) | 47 | 1.31 (0.253) |
|  | All isolates 1991-2018 | 168 (54) | 143 (46) | 311 | 1.01 (0.316) |
|  |  |  |  |  |  |
| B. Region | Lower North, SA | 15 (48) | 16 (52) | 31 | 0.02 (0.899) |
|  | Upper & Mid North, SA | 74 (52) | 68 (48) | 142 | 0.13 (0.722) |
|  | South East, SA | 29 (63) | 17 (37) | 46 | 1.59 (0.207) |
|  | VIC | 26 (67) | 13 (33) | 39 | 2.23 (0.135) |
|  | Yorke Peninsula, SA | 10 (36) | 18 (64) | 28 | 1.17 (0.280) |
|  | All main regions | 154 (54) | 132 (46) | 286 | 0.85 (0.357) |
|  |  |  |  |  |  |
| C. Year (PG-2) | 2015 | 16 (55) | 13 (45) | 29 | 0.16 (0.693) |
|  | 2016 | 17 (55) | 14 (45) | 31 | 0.15 (0.703) |
|  | 2017 | 17 (50) | 17 (50) | 34 | 0 (1.0) |
|  | 2018 | 18 (63) | 11 (38) | 29 | 0.86 (0.354) |
|  | PG-2 isolate (All years) | 68 (55) | 55 (45) | 123 | 0.69 (0.407) |
|  |  |  |  |  |  |
| D. Host | Farah / Farah AR | 16 (55) | 13 (45) | 29 | 0.16 (0.693) |
|  | Fiesta | 16 (57) | 12 (43) | 28 | 0.29 (0.592) |
|  | Nura / Nura AR | 5 (63) | 3 (38) | 8 | 0.25 (0.614) |
|  | PBA Rana | 8 (73) | 3 (27) | 11 | 1.20 (0.274) |
|  | PBA Samira | 13 (59) | 9 (41) | 21 | 0.36 (0.549) |
|  | PBA Zahra | 1 (25) | 3 (75) | 4 | 0.53 (0.465) |
|  | All commercial cultivars | 59 (58) | 43 (42) | 102 | 1.26 (0.261) |
