# Supplementary material for: Phenotypic and Genotypic Diversity of Ascochyta fabae Populations in Southern Australia
Source: Front Plant Sci. 2022 Aug 2;13:918211. doi: 10.3389/fpls.2022.918211 (PMC9380778; doi:10.3389/fpls.2022.918211)
Supplement: Supplementary file 1 [file Data_Sheet_1.zip › Supplementary Table 2.docx]

**Supplementary Table 2.** Population genetics statistical analysis. (A) Population genetics statistics for *A. fabae* populations. (B) Wright’s fixation index (F_st_) for pairwise comparisons. Pairwise F_st_ for SNP (grey shading) and SilicoDArT (no shading) genotypes for comparison of populations based on collection year and region, mating type, pathogenicity group and host.

A

| Year | 2014 | 2015 | 2016 | 2017 | 2018 |  |  |  |
| --- | --- | --- | --- | --- | --- | --- | --- | --- |
| 2014 | - | 0.004 | 0 | 0.003 | 0.003 |  |  |  |
| 2015 | 0.002 | - | 0.003 | 0.004 | 0.001 |  |  |  |
| 2016 | 0.001 | 0.002 | - | 0.003 | 0.002 |  |  |  |
| 2017 | 0.002 | 0.004 | 0.002 | - | 0.003 |  |  |  |
| 2018 | 0 | 0.001 | 0.001 | 0.001 | - |  |  |  |
| Region | YP | UN | MN | LN | SE | VIC |  |  |
| YP | - | 0.005 | 0.004 | 0.007 | 0.008 | 0.006 |  |  |
| UN | 0.003 | - | 0 | 0.002 | 0.004 | 0.001 |  |  |
| MN | 0.003 | 0.001 | - | 0.002 | 0.004 | 0.001 |  |  |
| LN | 0.004 | 0.002 | 0.002 | - | 0.006 | 0.003 |  |  |
| SE | 0.005 | 0.004 | 0.004 | 0.005 | - | 0.004 |  |  |
| VIC | 0.003 | 0.001 | 0.001 | 0.004 | 0.001 | - |  |  |
| Mating type | MAT1-1 | MAT1-2 |  |  |  |  |  |  |
| MAT1-1 | - | 0.001 |  |  |  |  |  |  |
| MAT1-2 | 0.002 | - |  |  |  |  |  |  |
| Pathogenicity group (PG) | PG-1 | PG-2 | PG-3 |  |  |  |  |  |
| Path1 | - | 0.002 | 0.003 |  |  |  |  |  |
| Path2 | 0.001 | - | 0 |  |  |  |  |  |
| Path3 | 0.001 | 0.001 | - |  |  |  |  |  |
| Host | Farah | Farah AR | Fiesta | Nura | Nura AR | PBA Rana | PBA Samira | PBA Zahra |
| Farah | - | 0.003 | 0.003 | 0.004 | 0 | 0.003 | 0.003 | 0.002 |
| Farah AR | 0.003 | - | 0.001 | 0.002 | 0 | 0.001 | 0 | 0 |
| Fiesta | 0.004 | 0 | - | 0.002 | 0 | 0.001 | 0.001 | 0 |
| Nura | 0.006 | 0.004 | 0.004 | - | 0 | 0 | 0.001 | 0 |
| Nura AR | 0.003 | 0 | 0.002 | 0 | - | 0 | 0 | 0 |
| PBA Rana | 0.003 | 0 | 0.001 | 0.002 | 0 | - | 0 | 0 |
| PBA Samira | 0.003 | 0 | 0.001 | 0.004 | 0 | 0 | - | 0 |
| PBA Zahra | 0.002 | 0 | 0.002 | 0.003 | 0 | 0.001 | 0.002 | - |

| **Population based on:** | **Population** | **^a^ N_ind_** | **^b^ H** | **^c^ H_exp_** | |
| --- | --- | --- | --- | --- | --- |
|  |  |  |  | **SNP** | **Silico** |
| Year | 2014 | 30 | 3.40 | 0.259 | 0.345 |
|  | 2015 | 44 | 3.78 | 0.251 | 0.337 |
|  | 2016 | 112 | 4.72 | 0.257 | 0.343 |
|  | 2017 | 42 | 3.74 | 0.246 | 0.339 |
|  | 2018 | 47 | 3.85 | 0.251 | 0.333 |
|  | Total | 275 | 5.62 | 0.255 | 0.343 |
| Region ^d^ | YP | 28 | 3.33 | 0.250 | 0.329 |
|  | UN | 15 | 2.71 | 0.247 | 0.335 |
|  | MN | 125 | 4.83 | 0.255 | 0.344 |
|  | LN | 23 | 3.14 | 0.262 | 0.347 |
|  | SE | 42 | 3.74 | 0.249 | 0.337 |
|  | VIC | 37 | 3.61 | 0.247 | 0.336 |
|  | Total | 270 | 5.60 | 0.255 | 0.343 |
| Mating type | MAT1-1 | 150 | 5.01 | 0.253 | 0.342 |
|  | MAT1-2 | 131 | 4.88 | 0.253 | 0.341 |
|  | Total | 281 | 5.64 | 0.254 | 0.343 |
| Pathogenicity group (PG) | PG-1 | 6 | 1.79 | 0.259 | 0.343 |
|  | PG-2 | 119 | 4.78 | 0.255 | 0.343 |
|  | PG-3 | 25 | 3.22 | 0.254 | 0.336 |
|  | Total | 150 | 5.01 | 0.255 | 0.342 |
| Host variety | Fiesta | 49 | 3.89 | 0.252 | 0.341 |
|  | Farah | 57 | 4.04 | 0.250 | 0.335 |
|  | Farah AR | 5 | 1.61 | 0.251 | 0.338 |
|  | PBA Rana | 21 | 3.04 | 0.250 | 0.343 |
|  | Nura | 12 | 2.48 | 0.258 | 0.340 |
|  | Nura AR | 3 | 1.10 | 0.273 | 0.335 |
|  | PBA Samira | 54 | 3.99 | 0.253 | 0.338 |
|  | PBA Zahra | 15 | 2.71 | 0.260 | 0.339 |
|  | Total | 216 | 5.38 | 0.254 | 0.341 |

B

^a^ number of individuals was equal to the number of MLGs;

^b^ H, Shannon-Weiner diversity index for SNP markers or SilicoDArT markers;

^c^ H_exp_, Nei’s unbiased gene diversity;

^d^ YP, Yorke Peninsula SA; UN, Upper North SA; MN, Mid North SA; LN, Lower North SA; SE, South East SA; VIC, Victoria
